# Adaptive filtering and smoothing algorithm based on variable structure interactive multiple model

**DOI:** 10.1038/s41598-023-39075-9

**Published:** 2023-08-10

**Authors:** Kai-Yu Hu, Jiaming Wang, Yuqing Cheng, Chunxia Yang

**Affiliations:** 1 The 304 Institute, China Aerospace Science and Industry Corporation, Beijing, 100074 People’s Republic of China; 2 Center for Applied Mathematics, China Aerospace Science and Industry Corporation, Beijing, 100074 People’s Republic of China

**Keywords:** Electrical and electronic engineering, Information technology

## Abstract

For maneuvering target tracking, a novel adaptive variable structure interactive multiple model filtering and smoothing (AVSIMMFS) algorithm is proposed in this paper. Firstly, an accurate model of the variable structure interactive multiple model algorithm is established. Secondly, by constructing a new model subset based on the original model subsets, the matching accuracy between the model subset and the actual maneuvering mode of the target is improved. Then, the AVSIMMFS algorithm is obtained by smoothing the filtered data of the new model subset. Because of the combination of forward filtering and backward smoothing, the target tracking accuracy is further improved. Finally, in order to verify the effectiveness of the algorithm, the simulation is carried out on two cases. The simulation results show that the tracking performance of AVSIMMFS algorithm is better than other methods and has lower calculation cost.

## Introduction

In the past few years, filters have been extensively employed in target tracking, parameter estimation, and state prediction^1–6^. When the target maneuvering is extremely complicated and the filtering model does not align with the target maneuvering model, the accuracy of the filtering methods based on a single model will significantly decrease. To address this problem, the interactive multiple model (IMM) algorithm exhibits superior performance^7–9^.

The IMM algorithm has received a great deal of attention in recent years, scholars have continuously developed and improved it in different aspects. In^10^, an IMM algorithm was designed based on the Constant Velocity (CV) and Current Statistics (CS) models, in which the average velocity of the CS model was first estimated by the least square method, and then the CS model was applied to the IMM algorithm, which improved the model accuracy. In^11^, IMM algorithm had been improved in many aspects, including the adoption of improved Kalman filter as a sub-filter, and an entropy-based model probability update formula. An alternative method to IMM had been proposed in^8,12^, the models in the model set were all composed of Constant accelerated (CA) models, which reduces the complexity of the model set. On the basis of this alternative method, the^13^ proposed an adaptive IMM algorithm, which firstly used the filter to estimate the acceleration of the target, and then selected the value near the estimated acceleration to build the model set, this method could reduce the number of models in the model set and improved the model accuracy while reducing the computational cost. In^14^, a second-order IMM algorithm was proposed based on the second-order Markov chain, which further improved the filtering accuracy due to the use of more prior information. Since the type and quantity of models in the model set of the above-mentioned IMM are unchanged, it is also called a Fixed Structure IMM (FSIMM)^15^.

In order to avoid accuracy errors due to model mismatch, when using the IMM algorithm, as many models as possible should be used to cover the target maneuvering model. However, it is worth noting that too many models in a single model set will also reduce the filtering accuracy^16,17^. To overcome this defect, a variety of Variable Structure IMM (VSIMM) algorithms have been presented. After continuous development and improvement, VSIMM can be divided into four types: Model-Group-Switching (MGS), Likely-Mode-Set (LMS), Expected-Mode-Augmentation (EMA) and Adaptive Grid (AG)^15,18,19^. Among them, MGS divides the model set into model subsets, only one model subset is selected for estimation at a time, and the switching between model subsets is selected according to the transition probability of model subsets^20^. The LMS divides the models into three types at each moment: impossible, important, and dominant models, and the model subset used for estimation at each moment consists of dominant and near-dominant models^21,22^. Similar to MGS, two typical estimators divide a large model set into small model subsets, then calculates the probabilities of all model subsets at the next moment, and selects the model subset with the highest probability for estimation^23^. Combined with graph theory, the AG algorithm forms a grid of all models, and uses prior information and current data to get a local refined grid, which forms a candidate model subset, and then selects models according to some rules to form a model subset for estimation at the next moment^24^.

It is worth noting that the above-mentioned EMA algorithm approximates the formula when calculating the likelihood function and model subset probability. To solve this problem, a normal VSIMM algorithm with accurate mathematical model is designed in this paper. In addition, considering that when EMA divides a large model set into small model subsets, in order to reduce the computational cost, although all model subsets include all models, they are not all permutations and combinations. Therefore, in order to ensure that there is a model subset that can best match the target maneuver mode in all model subsets, on the basis of the existing model subset, a new model subset is constructed according to the rules, thus obtaining the adaptive VSIMM (AVSIMM) algorithm. Because the model in the new model subset may match the target maneuver model better, the target tracking accuracy can be improved. Finally, based on the forward AVSIMM estimation, the data are further smoothed backward, so we get an AVSIMM Filtering and Smoothing (AVSIMMFS) algorithm, which can further improve the tracking accuracy. The main contributions of this study can be summarized as follows:

1. A model of normal VSIMM algorithm is established and applied in the design of filter, it has obvious effect on eliminating fast random clutter. The variable structure accurate model in the algorithm avoids EMA’s model approximation error and makes the subsequent filtering smooth more accurate.
2. Based on the VSIMM algorithm, the AVSIMM algorithm is presented and improves the matching degree between the model subset and the target maneuvering model, thus improving the tracking accuracy. The calculation time is greatly reduced by extracting the existing high probability models to form a new model subset and matching them directly.
3. The adaptive variable structure smoothing ensures that the peak buffeting of the tracking signal is eliminated when the target frequently switches flight modes, making the multi-target characteristics of the AVSIMMFS scheme more adaptive than the existing algorithms.

The rest of the paper is organized as follows. In Section “The IMM filtering and IMM smoothing algorithm”, the filtering and smoothing problems are described, and the mathematical models of the IMM filtering algorithm and the IMM smoothing algorithm are given. In Section “The AVSIMMFS algorithm”, the AVSIMMFS algorithm is designed based on normal VSIMM, including forward filtering and backward smoothing. The numerical simulation results are shown in Section “Simulation and discussion”. Eventually, the conclusion is summarized in Section “Conclusion”.

## The IMM filtering and IMM smoothing algorithm

### Filtering and smoothing problems

Assuming that the target may have $r$ motion models, the model set is denoted as $\Omega = \left\{ {M^{1} ,...,M^{r} } \right\}$, and the transition probability matrix between the models is:

1 $$P = \left( {\begin{array}{*{20}c} {p^{11} } & \cdots & {p^{1r} } \\ \vdots & \ddots & \vdots \\ {p^{r1} } & \cdots & {p^{rr} } \\ \end{array} } \right)$$

where, $p^{ij} (1 \le i \le r,1 \le j \le r)$ is the transition probability from model $i$ to model $j$.

The state equation for the discretization of the system is as follows:

2 $$x_{k}^{j} = F_{k}^{j} x_{k - 1}^{j} + w_{k - 1}^{j} ,j = 1,2,...,r$$

where, $x_{k}^{j}$ is the state vector of the system, $F_{k}^{j}$ is the state transition matrix of the model $j$, $w_{k - 1}^{j}$ is Gaussian white noise with mean 0, and its covariance matrix is $Q_{k - 1}^{j}$.

The measurement equation of model $j$ is:

3 $$z_{k} = H_{k}^{j} x_{k} + v_{k}^{j}$$

where, $z_{k}$ is the measurement vector, $H_{k}^{j}$ is the measurement matrix of model $j$, $v_{k}^{j}$ is Gaussian white noise with mean 0, and its covariance matrix is $R_{k}^{j}$.

Based on the above state equation, measurement equation and Bayesian theory, the IMM filtering algorithm estimates the system state $x_{k}$ at time $k$ according to the measurement set $Z^{k} = \left\{ {z_{1} ,...,z_{k} } \right\}$ and the model set $\Omega$. The purpose of smoothing is to estimate the system state $\hat{x}_{t|k}$ at the time $t$ according to all the current observations $Z^{k} = \left\{ {z_{1} ,z_{2} ,...,z_{k} } \right\}$, where $1 \le t \le k - 1$. Combining forward IMM filtering and backward IMM smoothing, the IMM Filtering and Smoothing algorithm (IMMFS) can be obtained.

### Forward IMM filtering

**Figure 1:**
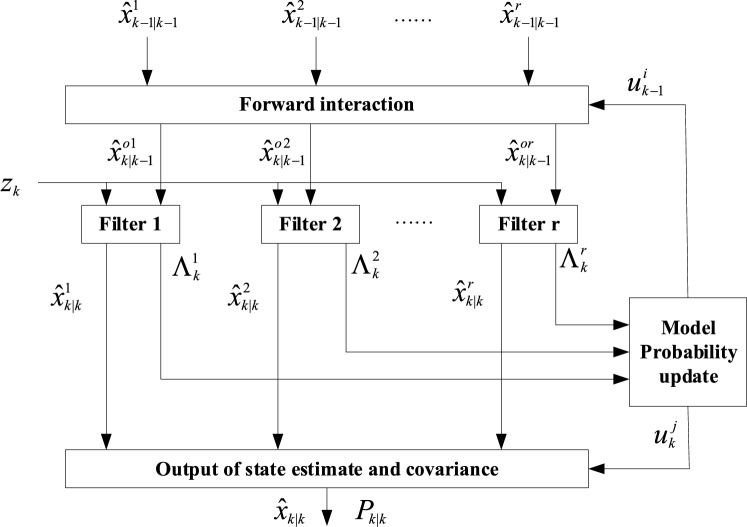
The forward IMM filtering algorithm.

The forward IMM filtering algorithm is described in Fig. 1, which can be divided into the following four steps^7,8^:

#### A_Step1: forward interaction

4 $$\hat{x}_{k - 1|k - 1}^{oj} = \sum\limits_{i = 1}^{r} {\hat{x}_{k - 1|k - 1}^{i} u_{k - 1|k - 1}^{ij} }$$

5 $$P_{k - 1|k - 1}^{oj} = \sum\limits_{i = 1}^{r} {u_{k - 1|k - 1}^{ij} \{ P_{k - 1|k - 1}^{i} } + [\hat{x}_{k - 1|k - 1}^{i} - \hat{x}_{k - 1|k - 1}^{oj} ][\hat{x}_{k - 1|k - 1}^{i} - \hat{x}_{k - 1|k - 1}^{oj} ]^{T} \}$$

where, $\hat{x}_{k - 1|k - 1}^{oj}$ and $P_{k - 1|k - 1}^{oj}$ are the mixed state estimation of model $j$ and the corresponding covariance matrix, respectively, $\hat{x}_{k - 1|k - 1}^{i}$ and $P_{k - 1|k - 1}^{i}$ are the Kalman estimate of model $i$ and the corresponding covariance matrix, and $u_{k - 1|k - 1}^{ij}$ is the mixing probability, and its calculation formula is:

6 $$u_{k - 1|k - 1}^{ij} = \frac{{p^{ij} u_{k - 1}^{i} }}{{c_{k}^{j} }}$$

where, $u_{k - 1}^{i}$ is the probability of model $i$, and the predicted probability $c_{k}^{j} = \sum\nolimits_{i = 1}^{r} {p^{ij} u_{k - 1}^{i} }$ is the normalization factor.

#### A_Step2: Kalman filtering

For each model, Kalman filtering is performed according to the following formulas:

7 $$\hat{x}_{k|k - 1}^{oj} = F_{k|k - 1}^{j} \hat{x}_{k - 1|k - 1}^{oj}$$

8 $$P_{k|k - 1}^{oj} = F_{k|k - 1}^{j} P_{k - 1|k - 1}^{oj} F_{k|k - 1}^{j\;\;\;\;\;T} + Q_{k - 1}^{j}$$

9 $$K_{k}^{oj} = P_{k|k - 1}^{oj} H_{k}^{jT} [H_{k}^{j} P_{k|k - 1}^{oj} H_{k}^{j} + R_{k}^{j} ]^{ - 1}$$

10 $$\hat{x}_{k|k}^{j} = \hat{x}_{k|k - 1}^{oj} + K_{k}^{oj} [z_{k} - H_{k}^{j} \hat{x}_{k|k - 1}^{oj} ]$$

11 $$P_{k|k}^{j} = [I - K_{k}^{oj} H_{k}^{j} ]P_{k|k - 1}^{oj}$$

#### A_Step3: model probability update

The probability of model $j$ at time $k$ is:

$$u_{k}^{j} = \frac{{\Lambda_{k}^{j} c_{k}^{j} }}{{\sum\limits_{i = 1}^{r} {\Lambda_{k}^{i} c_{k}^{i} } }}$$

where, $\Lambda_{k}^{j}$ obeys the Gaussian distribution, and its calculation formula is:

12 $$\Lambda_{k}^{j} = \frac{1}{{(2\pi )^{n/2} |S_{k}^{j} |^{1/2} }}\exp \left\{ { - \frac{1}{2}(\gamma_{k}^{j} )^{T} (S_{k}^{j} )^{ - 1} \gamma_{k}^{j} } \right\}$$

where,

13 $$\gamma_{k}^{j} = z_{k} - H_{k}^{j} \hat{x}_{k - 1|k - 1}^{oj}$$

14 $$S_{k}^{j} = H_{k}^{j} P_{k|k - 1}^{oj} H_{k}^{jT} + R_{k}^{j}$$

#### A_Step4: output

The state estimate and covariance of the forward IMM filtering algorithm are:

15 $$\hat{x}_{k|k} = \sum\limits_{j = 1}^{r} {\hat{x}_{k|k}^{j} u_{k}^{j} }$$

16 $$P_{k|k} = \sum\limits_{j = 1}^{r} {u_{k}^{j} \{ P_{k|k}^{j} + [\hat{x}_{k|k}^{j} } - \hat{x}_{k|k} ][\hat{x}_{k|k}^{j} - \hat{x}_{k|k} ]^{T} \}$$

### Backward IMM smoothing

**Figure 2:**
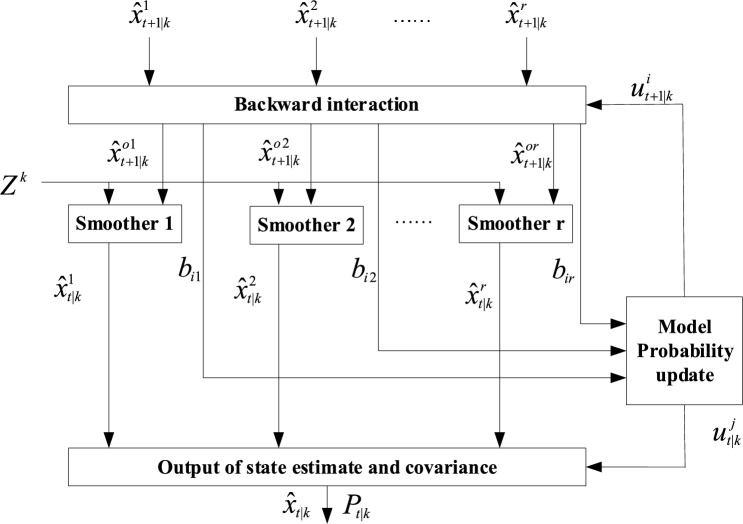
The backward IMM smoothing algorithm.

On the basis of the forward IMM filtering, the data is further smoothed to obtain the backward IMM smoothing algorithm. The block diagram of the IMM smoothing algorithm is shown in Fig. 2, including the following steps^25^:

#### B_step1: backward interaction

The backward state interaction and corresponding covariance are:

17 $$\hat{x}_{t + 1|k}^{oj} = \sum\limits_{i = 1}^{N} {u_{t + 1|k}^{i|j} \hat{x}_{t + 1|k}^{i} }$$

18 $$P_{t + 1|k}^{oj} = \sum\limits_{i = 1}^{N} {u_{t + 1|k}^{i|j} \{ P_{t + 1|k}^{i} + } [\hat{x}_{t + 1|k}^{i} - \hat{x}_{t + 1|k}^{oj} ][\hat{x}_{t + 1|k}^{i} - \hat{x}_{t + 1|k}^{oj} ]^{T} \}$$

where, $u_{t + 1|k}^{i|j}$ is the backward mixing probability, and its calculation formula is:

19 $$u_{t + 1|k}^{i|j} = \frac{1}{{d_{j} }}b_{ij} u_{t + 1|k}^{i}$$

where, $d_{j} = \sum\nolimits_{l = 1}^{N} {b_{lj} u_{t + 1|k}^{l} }$ is the normalization factor, $b_{ij}$ is the backward model transition probability, and its calculation formula is:

20 $$b_{ij} = \frac{1}{{e_{i} }}p_{ji} u_{t|t}^{j}$$

where, $e_{i} = \sum\nolimits_{l = 1}^{N} {p_{li} u_{t|t}^{l} }$ is the normalization factor.

#### B_Step2: Kalman smoothing^26^

The smoothing value and the corresponding covariance are:

21 $$\hat{x}_{t|k}^{j} = \hat{x}_{t|t}^{j} + A_{t|k}^{j} (\hat{x}_{t + 1|k}^{j} - \hat{x}_{t + 1|k}^{oj} )$$

22 $$P_{t|k}^{j} = P_{t|t}^{j} + A_{t|k}^{j} (P_{t + 1|k}^{j} - P_{t + 1|k}^{oj} )A_{t|k}^{j\;\;T}$$

where, $$A_{t|k}^{j} = P_{t|t}^{j} F_{t}^{jT} (P_{t + 1|k}^{oj} )^{ - 1}$$.

#### B_step3: model probability update

The model probability calculation formula after smoothing is:

23 $$u_{t|k}^{j} = \sum\limits_{i = 1}^{N} {b_{ij} u_{t + 1|k}^{i} }$$

#### B_step4: output

The state estimate and covariance of the backward IMM smoothing algorithm are:

24 $$\hat{x}_{t|k} = \sum\limits_{j = 1}^{N} {u_{t|k}^{j} \hat{x}_{t|k}^{j} }$$

25 $$P_{t|k} = \sum\limits_{j = 1}^{N} {u_{t|k}^{j} \left\{ {P_{t|k}^{j} + [\hat{x}_{t|k}^{j} - \hat{x}_{t|k} ][\hat{x}_{t|k}^{j} - \hat{x}_{t|k} ]^{T} } \right\}}$$

## The AVSIMMFS algorithm

### The normal VSIMM algorithm

**Figure 3:**
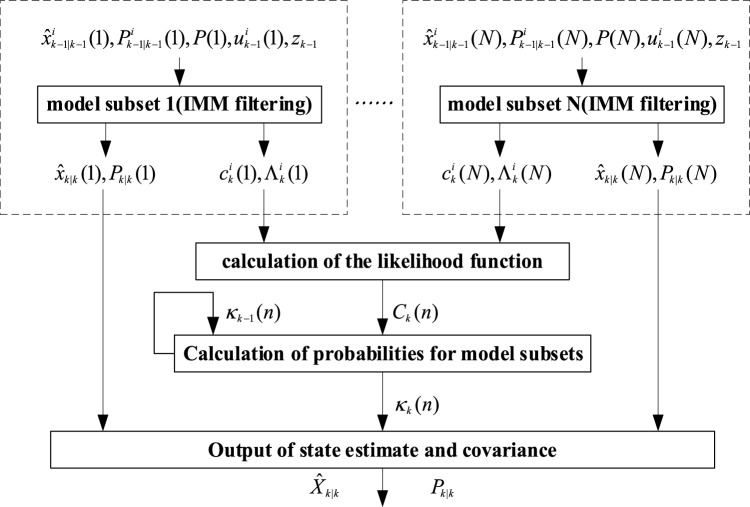
The NVSIMM algorithm.

In order to avoid too many models in the model set and reduce model errors, the normal VSIMM algorithm is designed in this section. The normal VSIMM filtering algorithm includes multiple model subsets, each model subset is independently used in parallel, and the estimation result of the model subset with the highest probability is selected as the final estimated state output. The block diagram of the normal VSIMM algorithm is shown in Fig. 3, which includes the following steps:

#### C_step1: parallel independent IMM filtering

For different model subsets, run the IMM algorithm independently to obtain the estimated state value $\hat{x}_{k|k} (n)$ and the corresponding covariance $P_{k|k} (n)$, and to obtain the model probability $u_{k}^{i} (n)$, likelihood function $\Lambda_{k}^{i} (n)$ and predicted probability $c_{k}^{i} (n)$ in each model subset, where $n$ is the number of the model subset, ${1} \le n \le N$, *i* is the number of the models in the model subset.

#### C_step2: calculation of the likelihood function for model subsets

26 $$p(\left. {z_{k} } \right|Z^{k - 1} ,\Pi_{k} (n)) = \sum\limits_{i} {p(\left. {z_{k} } \right|Z^{k - 1} ,M_{k}^{i} ,\Pi_{k} (n))p(\left. {M_{k}^{i} } \right|Z^{k - 1} ,\Pi_{k} (n))} = \sum\limits_{i} {\Lambda_{k}^{i} (n)c_{k}^{i} (n)}$$

where, $\Pi_{k} (n)$ represents the $n{\text{th}}$ model subset, $\Lambda_{k}^{i} (n)$ is calculated by A_Step3, and $c_{k}^{i} (n)$ is calculated by A_Step1. Let $C_{k} (n) = p(\left. {z_{k} } \right|Z^{k - 1} ,\Pi_{k} (n))$, then we have:

27 $$C_{k} (n) = \sum\limits_{i} {\Lambda_{k}^{i} (n)c_{k}^{i} (n)}$$

#### C_step3: calculation of probabilities for model subsets

Denote the model subset probability $p(\Pi_{k} (m)|Z^{k} ) = \kappa_{k} (n)$, then we have:

28 $$\kappa_{k} (n) = \frac{{p(z_{k} |Z^{k - 1} ,\Pi_{k} (n))p(\Pi_{k} (n)|Z^{k - 1} )}}{{\sum\limits_{n} {p(z_{k} |Z^{k - 1} ,\Pi_{k} (n))p(\Pi_{k} (n)|Z^{k - 1} )} }} = \frac{{p(z_{k} |Z^{k - 1} ,\Pi_{k} (n))p(\Pi_{k} (n)|Z^{k - 1} )}}{{p(z_{k} |Z^{k - 1} )}}$$

where, $p(\left. {z_{k} } \right|Z^{k - 1} ,\Pi_{k} (n))$ has been calculated in C_step2, the denominator is the normalization factor, and $p(\Pi_{k} (n)|Z^{k - 1} )$ according to the full probability formula is:

29 $$p(\Pi_{k} (n)|Z^{k - 1} ) = \sum\limits_{m} {p(\Pi_{k} (n)|\Pi_{k - 1} (m),Z^{k - 1} )p(\Pi_{k - 1} (m)|Z^{k - 1} )}$$

where $\kappa_{k - 1} (m) = p(\Pi_{k - 1} (m)|Z^{k - 1} )$ is the model subset probability at the previous moment. Applying the Markov property, it can be obtained that the model subset transition probability is independent of the observed value, namely:

30 $$p(\left. {\Pi_{k} (n)} \right|\Pi_{k - 1} (m),Z^{k - 1} ) = p(\left. {\Pi_{k + 1} (n)} \right|\Pi_{k} (m)) = \sum\limits_{j} {\prod\limits_{i} {p_{ij} } }$$

#### C_step4: output

The IMM estimation result of the model subset with the largest probability is selected as the final state estimation output. Firstly, the number of the model subset with the highest probability is calculated as follows:

31 $$n_{m} = \mathop {\max }\limits_{n} \{ \kappa_{k} (n)\}$$

Thus, the final state estimation and corresponding covariance can be obtained as follows:

32 $$\hat{x}_{k|k} = \hat{x}_{k|k} (n_{m} )$$

33 $$P_{k|k} = P_{k|k} (n_{m} )$$

### The AVSIMM algorithm

Assuming that the number of models in the model subset in the normal VSIMM algorithm is $L$, so $C_{r}^{L}$ model subsets can be obtained by permutation and combination. However, in order to reduce the computational cost, only $r - {1}$ model subsets are usually constructed to cover all models (denoted as the original model subset). These $r - {1}$ model subsets may not be the model subsets that best match the target maneuvering model. Therefore, in order to ensure that the calculation amount is not excessively increased, and to improve the matching degree between the model subsets and the target actual maneuvering model, a new model subset is constructed based on all the original model subsets, so we get the AVSIMM algorithm. The AVSIMM algorithm consists of the following steps:

#### D_step1: parallel independent IMM filtering

Operate on all original model subsets in the same way as C_step1.

#### D_step2: calculation of the likelihood function for model subsets

Operate on all original model subsets in the same way as C_step2.

#### D_step3: calculation of probabilities for model subsets

Operate on all original model subsets in the same way as C_step3.

#### D_step4: build a new model subset

Find the *L* model subsets with the highest probability according to the following formula:

34 $$\left\{ {\Pi_{k + 1} (m_{1} ), \cdots ,\Pi_{k + 1} (m_{L} )} \right\} = {\text{maxL}}\left\{ {p(\Pi_{k + 1} (1)|Z^{k} ), \cdots ,p(\Pi_{k + 1} (N)|Z^{k} )} \right\}$$

where, the symbol ${\text{maxL}}$ is used to find the model subsets with the 1st, 2nd, …, Lth largest probability according to the set $\left\{ {p(\Pi_{k + 1} (1)|Z^{k} ), \ldots ,p(\Pi_{k + 1} (N)|Z^{k} )} \right\}$, which means $p(\Pi_{k + 1} (m_{L} )|Z^{k} ) \le \cdots \le p(\Pi_{k + 1} (m_{1} )|Z^{k} )$. Then, the model with the highest probability is selected from each model subset to form a new model subset. If the new model subset has the same model, or is the same as the original model subset, skip to D_step8; otherwise, continue to the next step.

#### D_step5: parallel independent IMM filtering

Operate on all original model subsets and new model subset in the same way as C_step1, the sum of the number of original model subsets and new model subsets is denoted by $N^{\prime} = N + L$.

#### D_step6: calculation of the likelihood function for model subsets

Operate on all original model subsets and new model subset in the same way as C_step2.

#### D_step7: calculation of probabilities for model subsets

Operate on all original model subsets and new model subset in the same way as C_step3.

#### D_step8: output

The procedure for this step is the same as C_step4.

### The AVSIMMFS algorithm

**Figure 4:**
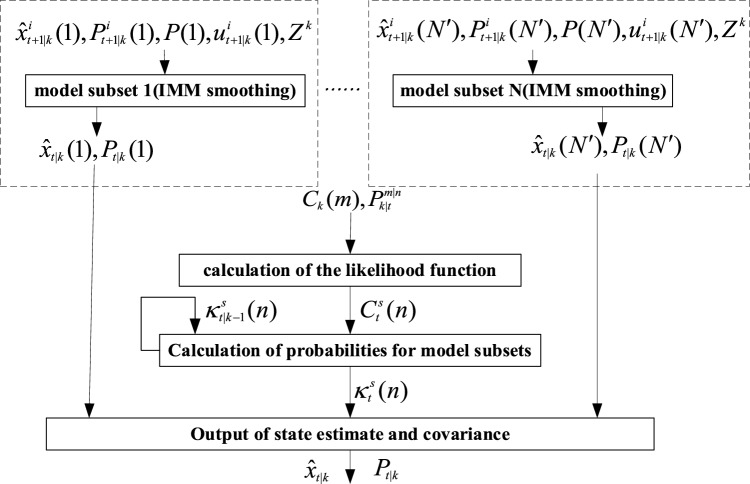
Backward smoothing process.

In this section, the AVSIMMFS algorithm can be obtained by further backward smoothing for the data obtained by forward AVSIMM filtering. The smoothing process is shown in Fig. 4, including the following steps:

#### E_step1: parallel independent IMM smoothing

$N^{\prime}$ original model subsets and new model subsets are operated independently, and the backward IMM smoothing algorithm was run respectively to obtain the smoothing estimate $\hat{x}_{t|k} (n)$ and the corresponding covariance matrix $P_{t|k} (n)$.

#### E_step2: Calculation of the likelihood function for model subsets

Applying the full probability formula and the Markov property, the likelihood function of the model subset can be obtained as:

35 $$\begin{aligned} p(\left. {z_{k} } \right|Z^{k - 1} ,\Pi_{t} (n)) &= \sum\limits_{m} {p(\left. {z_{k} } \right|Z^{k - 1} ,\Pi_{k} (m),\Pi_{t} (n))p(\left. {\Pi_{k} (m)} \right|Z^{k - 1} ,\Pi_{t} (n))} \\ &= \sum\limits_{m} {p(\left. {z_{k} } \right|Z^{k - 1} ,\Pi_{k} (m))p(\left. {\Pi_{k} (m)} \right|Z^{k - 1} ,\Pi_{t} (n))} \\ & = \sum\limits_{m} {p(\left. {z_{k} } \right|Z^{k - 1} ,\Pi_{k} (m))p(\left. {\Pi_{k} (m)} \right|\Pi_{t} (n))} \end{aligned}$$

where, $p(\left. {z_{k} } \right|Z^{k - 1} ,\Pi_{k} (m))$ is the likelihood function of the model subset in the forward NVSIMM filtering process. Applying the full probability formula and the Markov property, $p(\left. {\Pi_{k} (m)} \right|\Pi_{t} (n))$ can be transformed into:

36 $$\begin{aligned} &p(\left. {\Pi_{k} (m)} \right|\Pi_{t} (n)) \\ &\quad = \sum\limits_{{n_{t + 1} }} {p(\left. {\Pi_{k} (m)} \right|\Pi_{t + 1} (n_{t + 1} ),\Pi_{t} (n))p(\left. {\Pi_{t + 1} (n_{t + 1} )} \right|\Pi_{t} (n))} \\ &\quad = \sum\limits_{{n_{t + 1} }} {p(\left. {\Pi_{k} (m)} \right|\Pi_{t + 1} (n_{t + 1} ))p(\left. {\Pi_{t + 1} (n_{t + 1} )} \right|\Pi_{t} (n))} \\ &\quad = \sum\limits_{{n_{t + 1} }} {\left( {\sum\limits_{{n_{t + 2} }} {p(\left. {\Pi_{k} (m)} \right|\Pi_{t + 2} (n_{t + 2} ))p(\left. {\Pi_{t + 2} (n_{t + 2} )} \right|\Pi_{t + 1} (n_{t + 1} ))} } \right)p(\left. {\Pi_{t + 1} (n_{t + 1} )} \right|\Pi_{t} (n))} \\&\quad = \cdots \cdots \\ &\quad = \sum\limits_{{n_{t + 1} }} {\left\{ {\sum\limits_{{n_{t + 2} }} {\left( { \cdots \sum\limits_{{n_{k - 1} }} {p(\left. {\Pi_{k} (m)} \right|\Pi_{k - 1} (n_{k - 1} ))p(\left. {\Pi_{k - 1} (n_{k - 1} )} \right|\Pi_{k - 2} (n_{k - 2} )) \cdots } } \right)p(\left. {\Pi_{t + 2} (n_{t + 2} )} \right|\Pi_{t + 1} (n_{t + 1} ))} } \right\}}\\ &\qquad \times p(\left. {\Pi_{t + 1} (n_{t + 1} )} \right|\Pi_{t} (n)) \\ \end{aligned}$$

where, $p(\left. {\Pi_{t + 1} (n_{t + 1} )} \right|\Pi_{t} (n)),...,p(\left. {\Pi_{k} (m)} \right|\Pi_{k - 1} (n_{k - 1} ))$ is the transition probability of the model subset, which has been calculated in C_step3, and the model subset transition probability $p(\left. {\Pi_{k} (m)} \right|\Pi_{k - 1} (n_{k - 1} )$ is denoted by ${\rm P}_{k|k - 1}^{{m|n_{k - 1} }}$, then formula (36) becomes:

37 $${\rm P}_{k|t}^{m|n} = \sum\limits_{{n_{t + 1} }} {\left\{ {\sum\limits_{{n_{t + 2} }} {\left( { \cdots \sum\limits_{{n_{k - 1} }} {{\rm P}_{k|k - 1}^{{m|n_{k - 1} }} {\rm P}_{k - 1|k - 2}^{{n_{k - 1} |n_{k - 2} }} \cdots } } \right){\rm P}_{t + 2|t + 1}^{{n_{t + 2} |n_{t + 1} }} } } \right\}} {\rm P}_{t + 1|t}^{{n_{t + 1} |n_{t} }}$$

Denote $p(\left. {z_{k} } \right|Z^{k - 1} ,\Pi_{t} (n))$ as $C_{t}^{s} (n)$, then Eq. (35) becomes:

38 $$C_{t}^{s} (n) = \sum\limits_{m} {C_{k} (m){\rm P}_{k|t}^{m|n} }$$

#### E_step3: calculation of probabilities for model subsets

According to Bayes theorem, the model subset probability can be obtained as:

39 $$p(\Pi_{t} (n)|Z^{k} ) = \frac{{p(\left. {z_{k} } \right|Z^{k - 1} ,\Pi_{t} (n))p(\Pi_{t} (n)|Z^{k - 1} )}}{{\sum\limits_{n} {p(\left. {z_{k} } \right|Z^{k - 1} ,\Pi_{t} (n))p(\Pi_{t} (n)|Z^{k - 1} )} }}$$

where, $p(\Pi_{t} (n)|Z^{k - 1} )$ has been calculated in the smoothing process at the previous moment and is a known value. $p(\left. {z_{k} } \right|Z^{k - 1} ,\Pi_{t} (n))$ is obtained in E_step2. Denoting $p(\Pi_{t} (n)|Z^{k} )$ as $\kappa_{t|k}^{s} (n)$, Eq. (39) can be transformed into:

40 $$\kappa_{t|k}^{s} (n) = \frac{{p(\left. {z_{k} } \right|Z^{k - 1} ,\Pi_{t} (n))\kappa_{t|k - 1}^{s} (n)}}{{\sum\limits_{n} {p(\left. {z_{k} } \right|Z^{k - 1} ,\Pi_{t} (n))\kappa_{t|k - 1}^{s} (n)} }}$$

#### E_step4: output

The model subset number with the highest probability is:

41 $$n_{m}^{s} = \mathop {\max }\limits_{n} \{ \kappa_{t|k}^{s} (n)\}$$

The state estimate and the corresponding covariance of AVSIMMFS are:

42 $$\hat{x}_{t|k} = \hat{x}_{t|k} (n_{m}^{s} )$$

43 $$P_{t|k} = P_{t|k} (n_{m}^{s} )$$

It is worth noting that applying the smoothing process to normal VSIMM, the normal VSIMM filtering and smoothing algorithm can also be obtained. In the normal VSIMM filtering and smoothing algorithm, the smoothing process only operates on the $N$ original model subsets; in AVSIMMFS algorithm, the smoothing process needs to operate on the $N^{\prime}$ original model subsets and the new model subsets.

With the development of semiconductor technology, the computing speed of multi-core heterogeneous chips on aircraft will become faster and faster^27–29^. Through the distributed computing technology of the on-board computer, different algorithms can be injected into different chip cores or kernel computing units, and then parallel computing. Therefore, complex algorithm is naturally fast. Based on the advanced hardware resources, we complete the design process in steps: Step 1: forward IMM and a backward IMM. Step 2: a model of normal VSIMM algorithm is established and applied. Step 3: based on the VSIMM algorithm, the AVSIMM algorithm is presented and improved the matching degree between the model subset and the target model. When we have completed the first step of IMM, we first conduct validity verification experiment, rather than rushing to design Step 2. Under the condition of clear verification criteria, the Step 1 of successful verification is used to design the VSIMM in Step 2: the process of connecting multiple IMM combinations in parallel and calculating the largest possible subset. The Step 2 of the design also needs to be verified first rather than rushed into the third step of the content. Similarly, the Step 2 of verifying success is used as the basis for completing the Step 3, which adds D_step4 to the Step 2. This improvement avoids a lot of repeated calculation of multiple subsets, and only needs to calculate each subset once and get a new subset, then use the new subset to perform filtering and smoothing task, which theoretically ensures the rapidity of the algorithm. Finally, the experiment is verified again to ensure the whole scheme feasibility. By independently verifying/designing each step, adding D_step4, this study ensures the complexity, feasibility and fast real-time of the algorithm scheme from both experimental and method design.

### Simulation and discussion

In order to verify the effectiveness of AVSIMMFS, this section first analyzes three cases. The first and third cases are simulation data, and the second case is real data. Suppose the target maneuvers in a two-dimensional plane.

In the first case, the initial position, velocity and acceleration of the target are (300 m, 100 m), (5 m/s, 0) and (0, 0), respectively. The maneuvering parameters of the target are as follows:

1. CV motion in 20 s.
2. CT motion in 20 s, the turning angle rate is 0.157 rad/s.
3. CA motion in 10 s, its acceleration is (− 2 m/s^2^, 0 m/s^2^).
4. CT motion in 10 s, the turning angle rate is 0.157 rad/s.

In the second case, the initial position, velocity and acceleration of the target are (30 km, 40 km), (300 m/s, 0), (0, 0), respectively. The maneuvering parameters of the target are as follows:

1. Singer motion^30^ in 30 s, its acceleration is (− 20 m/s^2^, − 20 m/s^2^), maneuvering constant *α* = 1/60.
2. Both CV motions in 30 s.
3. CA motion in 30 s, its acceleration is (20 m/s, 10).
4. CT motion in 30 s, the turning angle rate is 0.2 rad/s.

**Figure 5:**
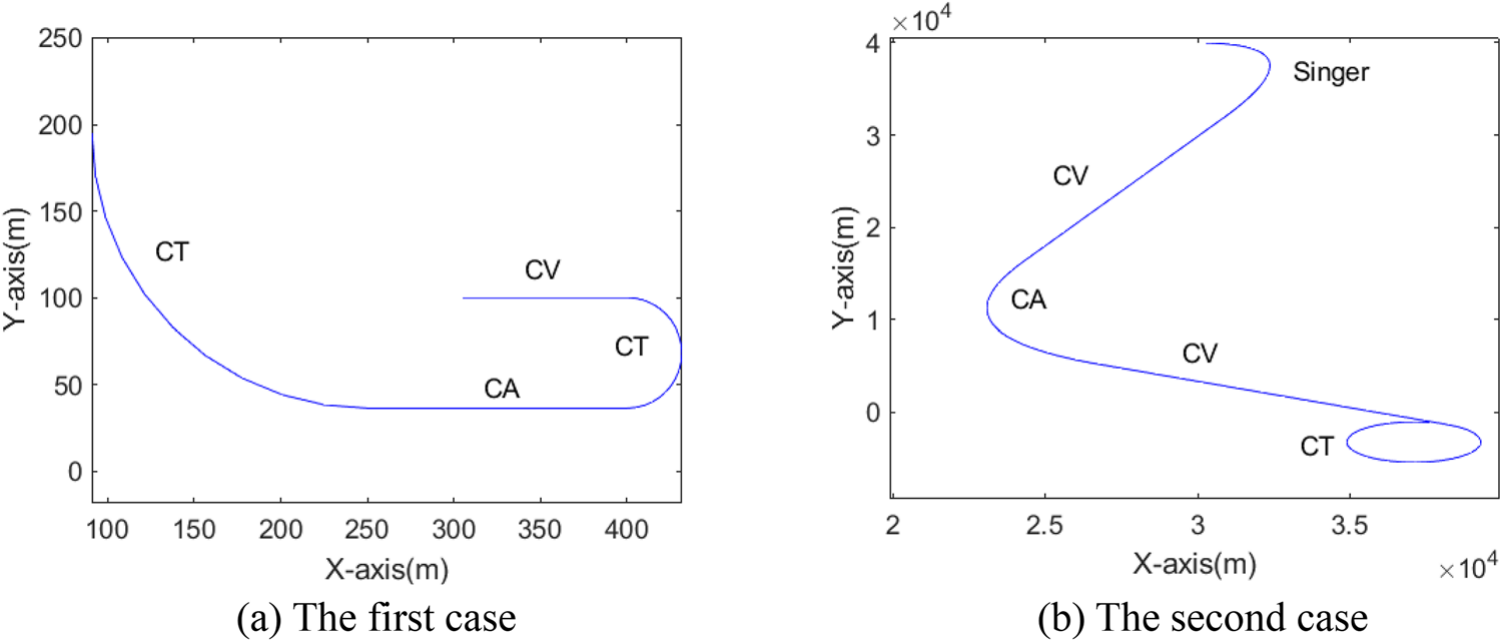
The target trajectories in two cases.

In the two cases, the target's trajectory is shown in Fig. 5.

The performance of IMM, normal VSIMM, AVSIMM and AVSIMMFS is analyzed and compared by Monte Carlo method. For the first and second cases, the model set of all algorithms is $\left\{ {{\text{CV}},{\text{CA}},{\text{CT}}} \right\}$, normal VSIMM, AVSIMM and AVSIMMFS algorithms include two model subsets, $\left\{ {{\text{CV}},{\text{CA}}} \right\}$ and $\left\{ {{\text{CV}},{\text{CT}}} \right\}$, respectively. For the second case, the model set of all algorithms is $\left\{ {{\text{CV}},{\text{CA}},{\text{CT}},{\text{Singer}}} \right\}$, normal VSIMM, AVSIMM and AVSIMMFS algorithms include three model subsets, $\left\{ {{\text{CV}},{\text{CA}}} \right\}$, $\left\{ {{\text{CV}},{\text{CT}}} \right\}$ and $\left\{ {{\text{CV}},{\text{Singer}}} \right\}$. The model transition probability matrixes of three models and four models are:

$$P_{3} = \left( {\begin{array}{*{20}c} {0.95} & {0.025} & {0.025} \\ {0.025} & {0.95} & {0.025} \\ {0.025} & {0.025} & {0.95} \\ \end{array} } \right),\;\;\;P_{4} = \left( {\begin{array}{*{20}c} {0.94} & {0.02} & {0.02} & {0.02} \\ {0.02} & {0.94} & {0.02} & {0.02} \\ {0.02} & {0.02} & {0.94} & {0.02} \\ {0.02} & {0.02} & {0.02} & {0.94} \\ \end{array} } \right)$$

**Figure 6:**
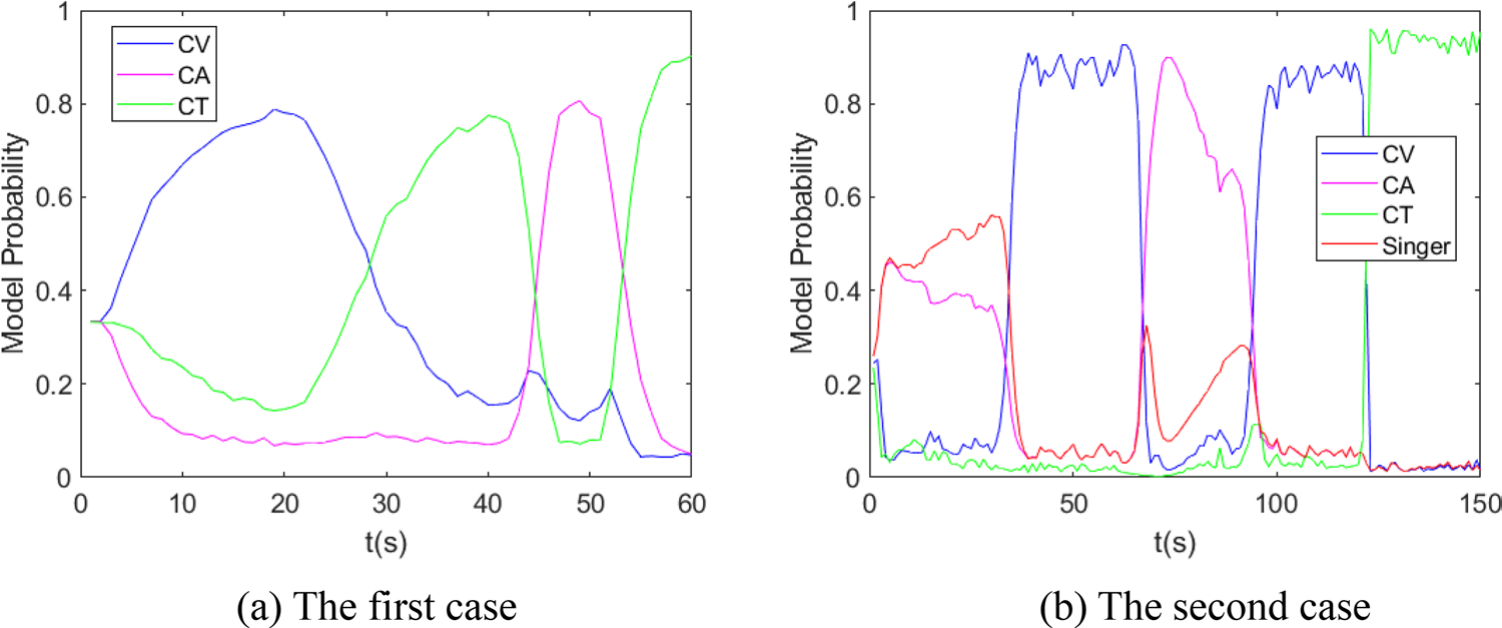
Model probability in IMM algorithm.

**Figure 7:**
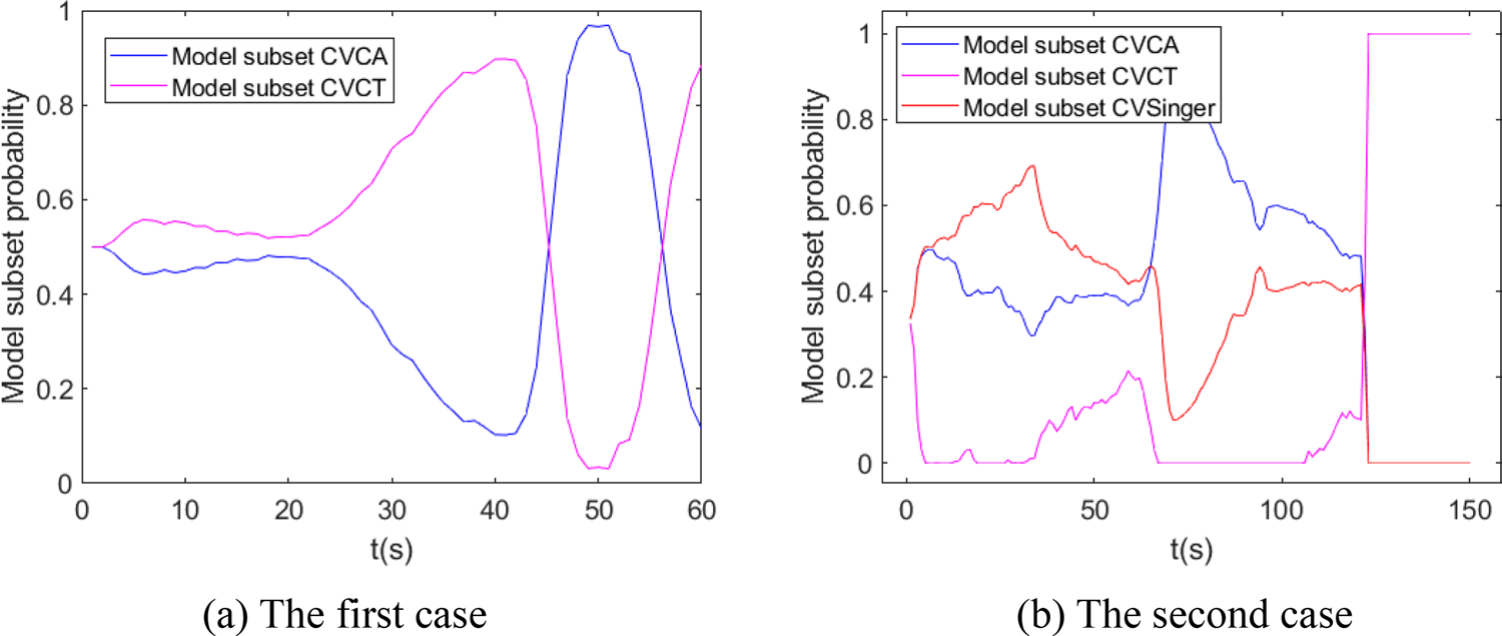
Model subset probability in normal VSIMM algorithm.

**Figure 8:**
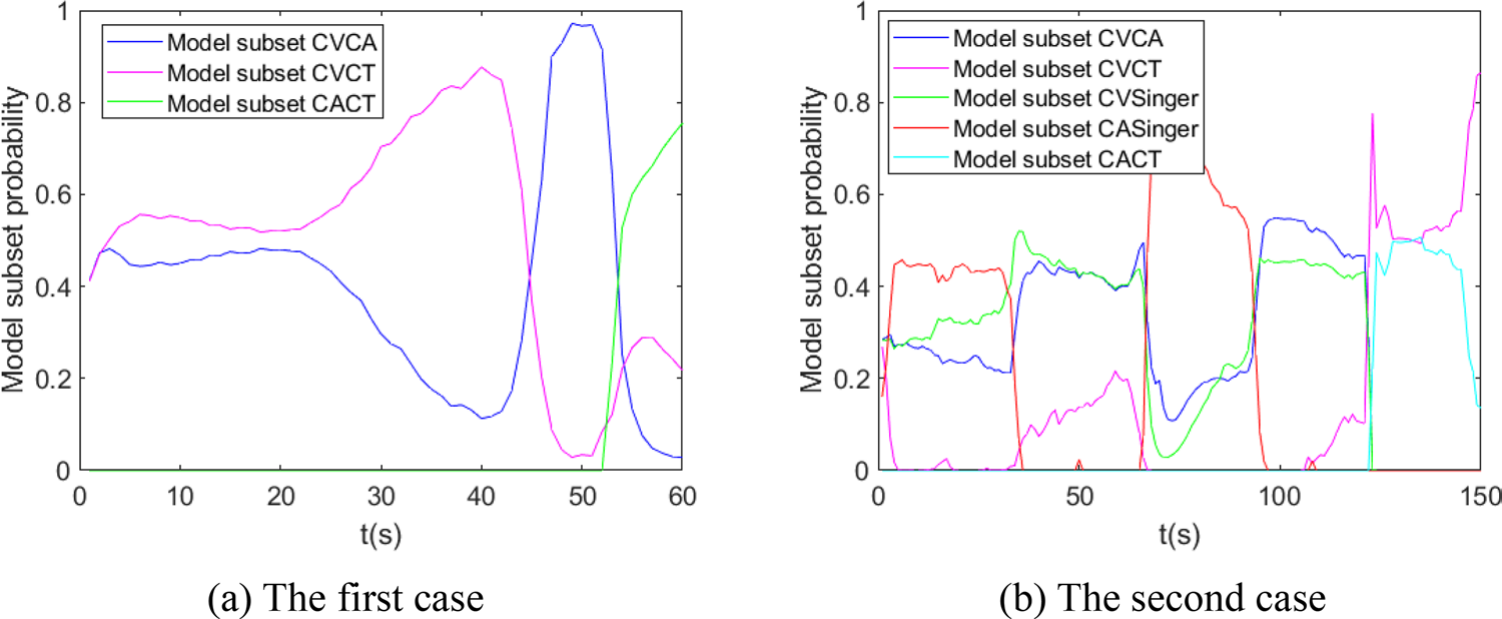
Model subset probability in AVSIMM algorithm.

**Figure 9:**
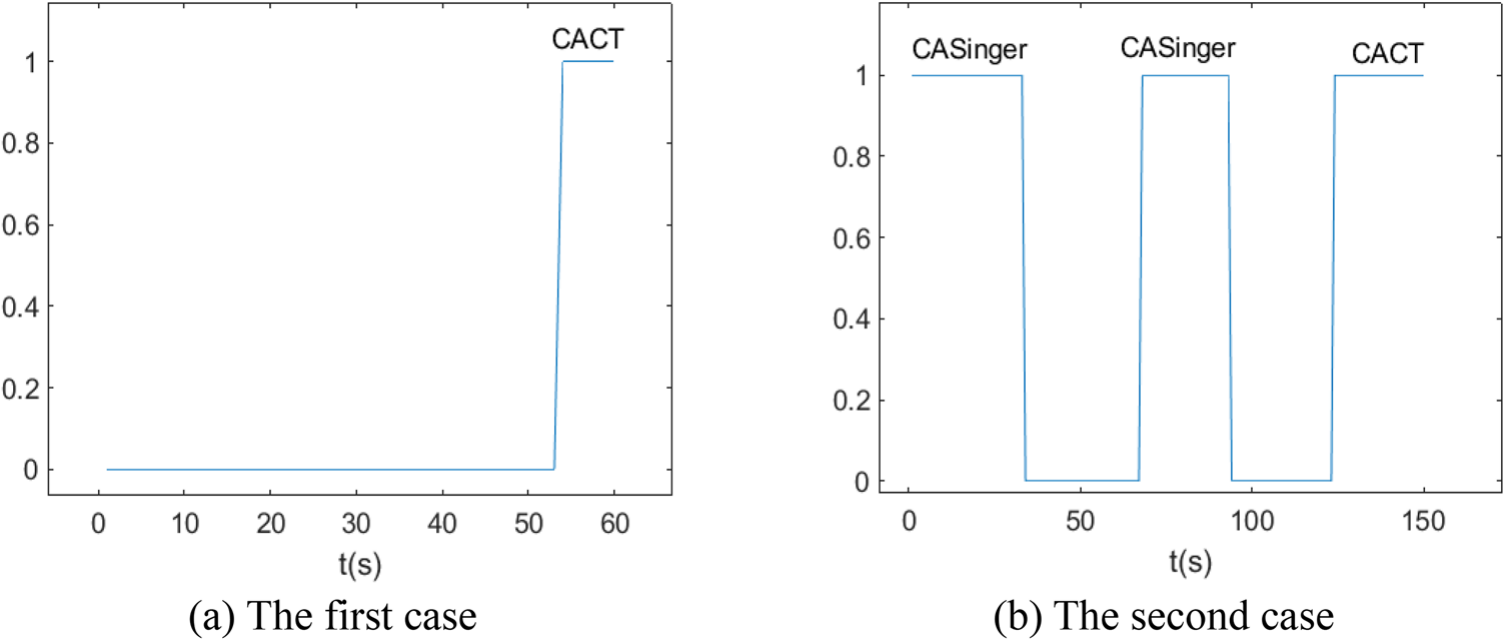
Whether a new model subset CAVT is built in AVSIMM.

**Figure 10:**
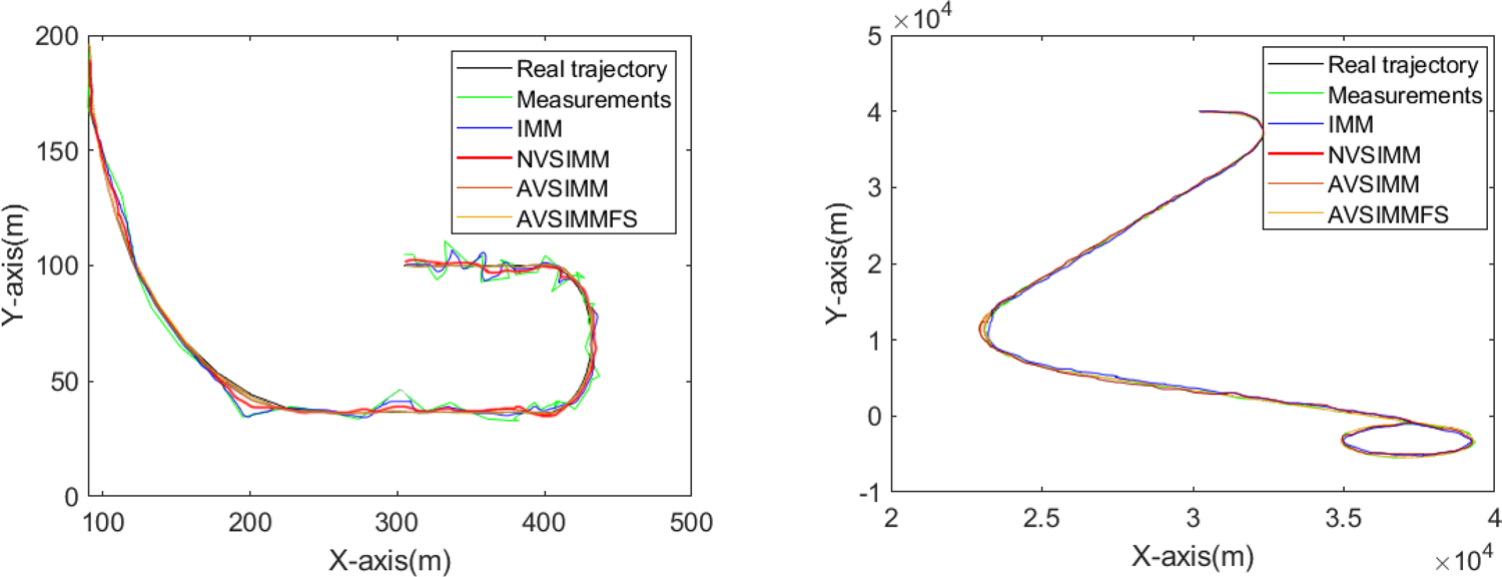
Target trajectory tracking effect.

**Figure 11:**
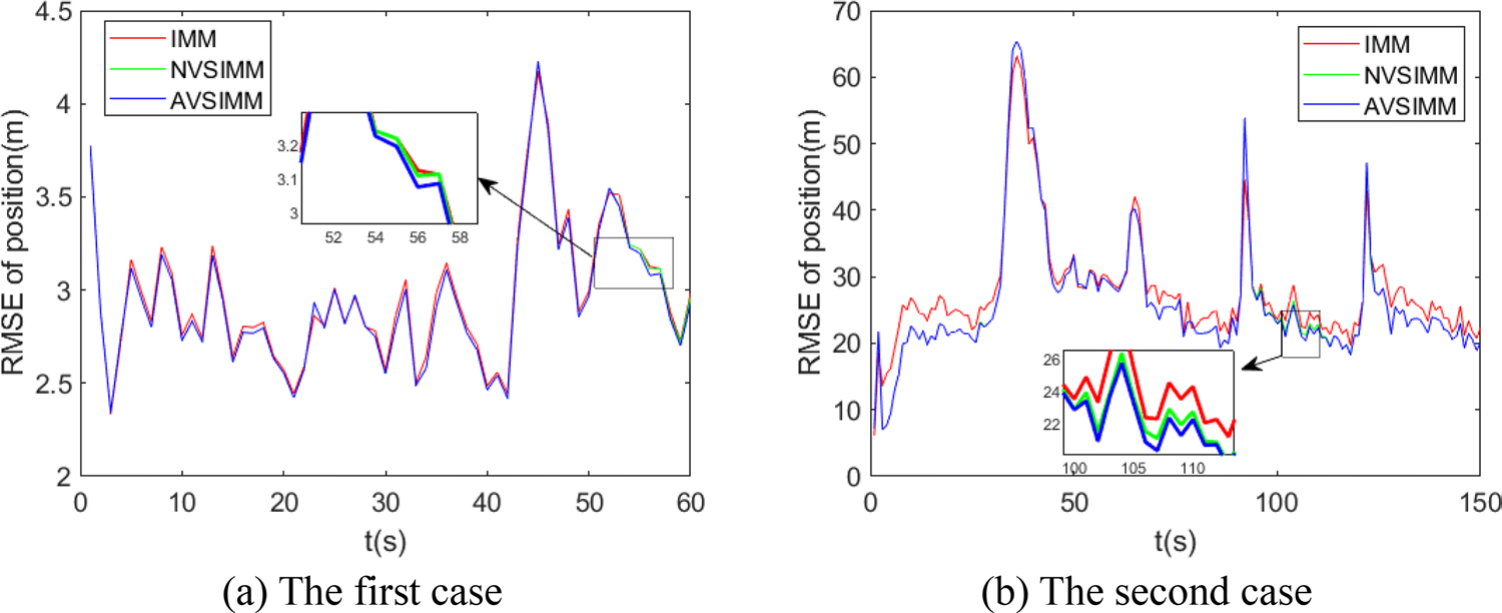
Root mean square error (RMSE) of position tracking.

**Figure 12:**
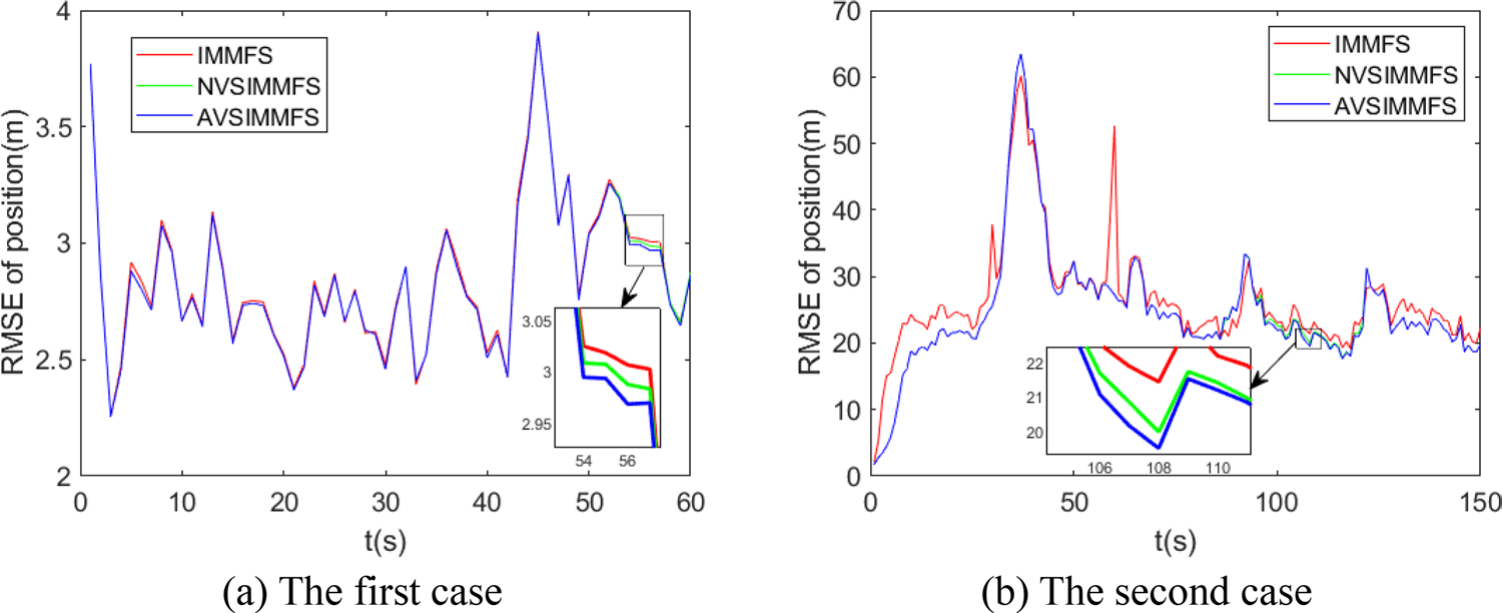
RMSE of position for IMMFS, normal VSIMMFS and AVSIMMFS.

**Figure 13:**
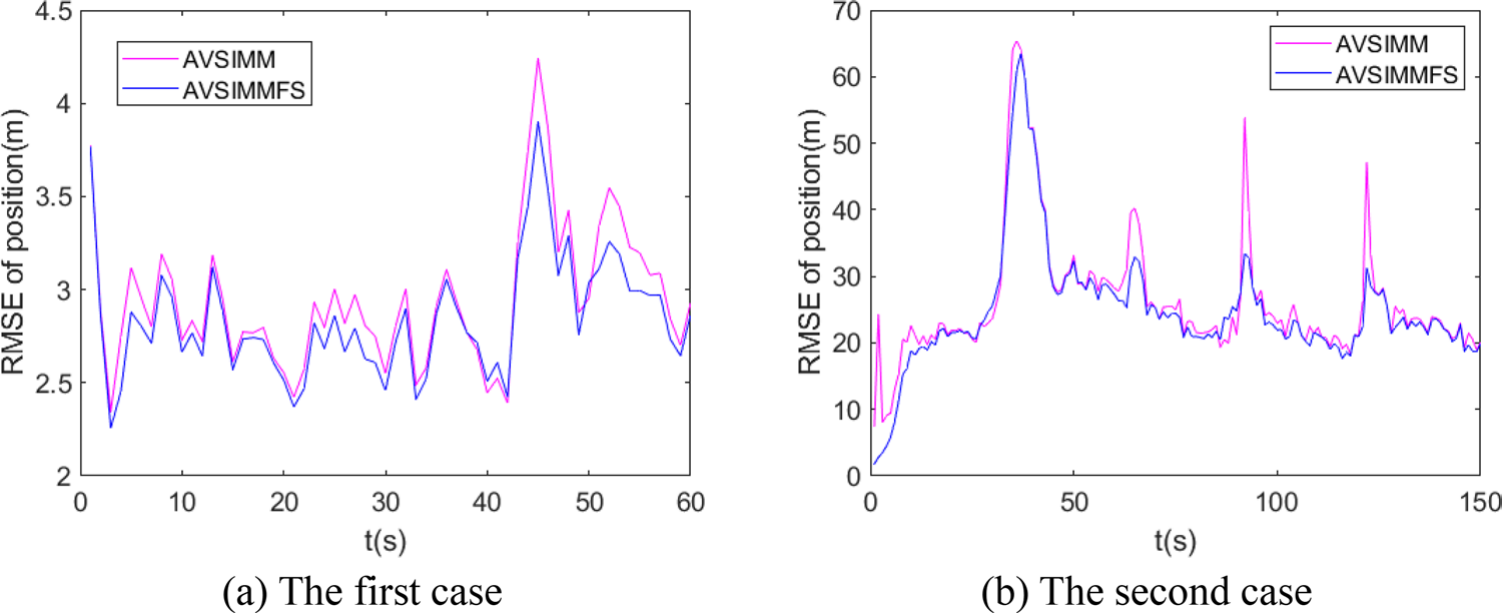
RMSE of position for AVSIMM and AVSIMMFS.

In the first case and the third case, it is assumed that the standard deviations of the system process noise and the measurement noise are 1 m/s^2^ and 50 m, respectively. The values of the covariance $Q_{k - 1}^{j}$ are between 0 and 0.03. It is randomly designed and calculated without affecting the result. The measured data of the second case are obtained by GPS measurement. The simulation results are shown in Figs. 6, 7, 8, 9, 10, 11, 12, and 13.

Figure 6 shows the model probability curve in the IMM algorithm. For these two cases, the model corresponding to the curve with the highest probability is the same as the target actual maneuvering model. Figure 7 shows the probability curve of model subsets in normal VSIMM, in which the model subset with the largest model probability always includes the model that matches the actual maneuvering model of the target.

It can be seen from Fig. 8 that the model subset probability curve in AVSIMM is roughly the same as that in Fig. 7, and the main difference between AVSIMM and normal VSIMM lies in the AVSIMM algorithm builds a new model subset. From Fig. 8a, it can be seen that AVSIMM constructs a new model subset $\left\{ {{\text{CA}},{\text{CT}}} \right\}$ when the target performs CT maneuver around 50–60 s. It can be seen from Fig. 8b that a new model subset $\left\{ {{\text{CA}},{\text{Singer}}} \right\}$ is constructed when the target performs Singer maneuver in 0–30 s and CA maneuver in 60–90 s. For all of the above cases, the probability of the newly constructed model subset is greater than that of the original model subset, because the newly constructed model subset matches the target actual maneuvering model more closely. However, for the third case, when the target performs CT maneuver around 120–150 s, a new model subset $\left\{ {{\text{CA}},{\text{CT}}} \right\}$ is constructed. At this time, the probability of the new model subset $\left\{ {{\text{CA}},{\text{CT}}} \right\}$ is less than the probability of the original model subset $\left\{ {{\text{CV}},{\text{CT}}} \right\}$, so the estimation of the new model subset will not be the final estimation of the AVSIMM algorithm. Figure 9 depicts the moment when the new model subset is constructed. Analyzing this figure leads to the same conclusion as Fig. 8.

As can be seen from Fig. 10, IMM, normal VSIMM, AVSIMM and AVSIMMFS all have good target tracking capabilities, and the differences among them are reflected in Figs. 11, 12, and 13.

Even considering only the results of Fig. 10b, Table 1 still shows the difference in experimental results. In fact, due to the larger scale, the true errors are even larger than that of Fig. 10a. Table 1 is as follows:

**Table 1:** Comparative analysis of tracking errors in different algorithm design stages.

	IMM	NVSIMM	AVSIMM	AVSIMMFS
e_av_/m				
Case 1	5.7	1.4	0.83	0.42
Case 2	49	17	13	3
e_max_/m				
Case 1	9.8	2.9	1.5	0.51
Case 2	241	58	20	7

In Table 1, e_av_ and e_max_ are the average error and maximum error respectively. It can be seen from the table that the error differentiation in different algorithm design stages in Fig. 10 indicates that the AVSIMMFS designed at last has the best performance based on the previous design. This ensures that the readers can distinguish between the filtering and smoothing capabilities in Fig. 10.

Figures 11, 12, and 13 shows the RMSE of different algorithms for target position tracking. From Fig. 11, it can be seen that the RMSE of normal VSIMM and AVSIMM is smaller than that of IMM, and only for a short time when the target maneuver mode is changed, the performance of normal VSIMM and AVSIMM will decrease. The performance of normal VSIMM and AVSIMM is very close. Main difference between the two is in the time period when the new model subset is built. As can be seen from the enlarged area in Fig. 11, the performance of AVSIMM is slightly better than that of normal VSIMM, because the new constructed model subset better matches the actual maneuvering model of the target. ‘NVSIMM’ means normal VSIMM.

Figure 12 compares the tracking performance of IMMFS, normal VSIMMFS and AVSIMMFS. It can be seen from the figure that the RMSE of AVSIMMFS and normal VSIMMFS for target tracking is less than the RMSE of IMMFS. Similar to the results in Fig. 11, the performance of AVSIMMFS and normal VSIMMFS is similar, but the main difference between them is that AVSIMMFS performs slightly better than normal VSIMMFS in the time period when the new model subset is built. Figure 13 analyzes and compares AVSIMM and AVSIMMFS. It can be seen from the figure that the RMSE of AVSIMMFS for position estimation is smaller than that of AVSIMM, so the AVSIMMFS has better performance.

**Figure 14:**
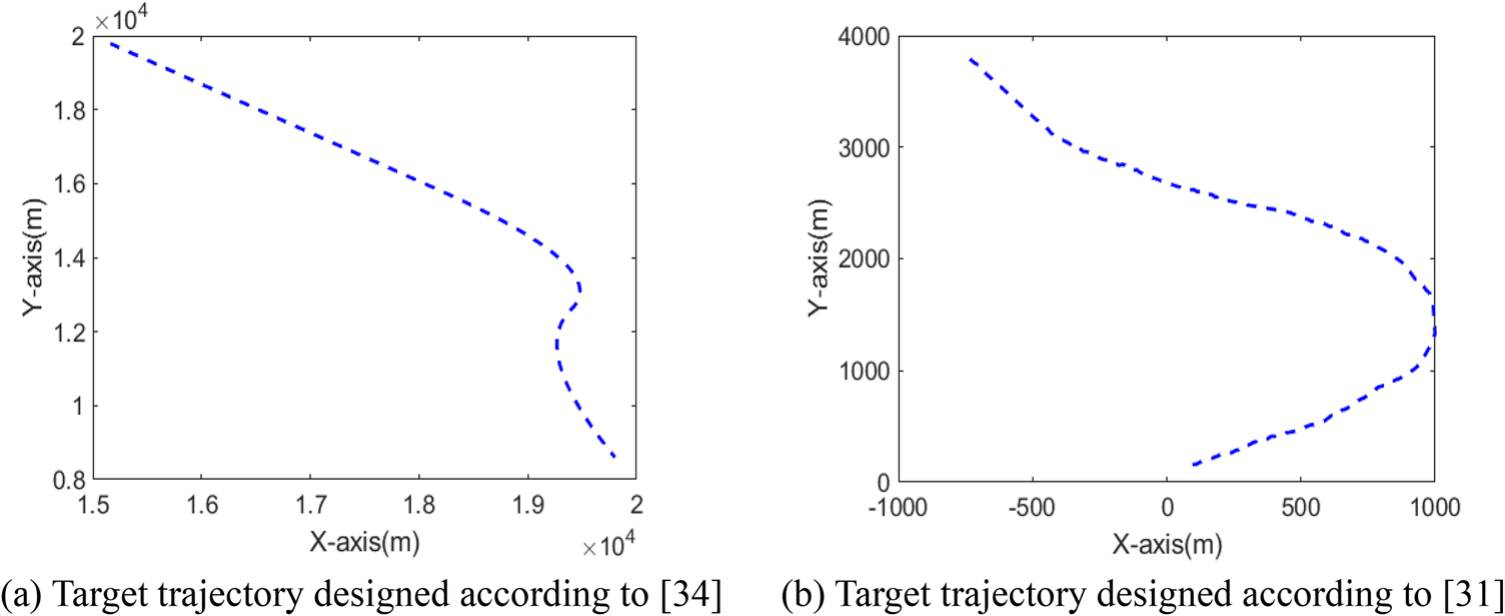
Target trajectories.

**Figure 15:**
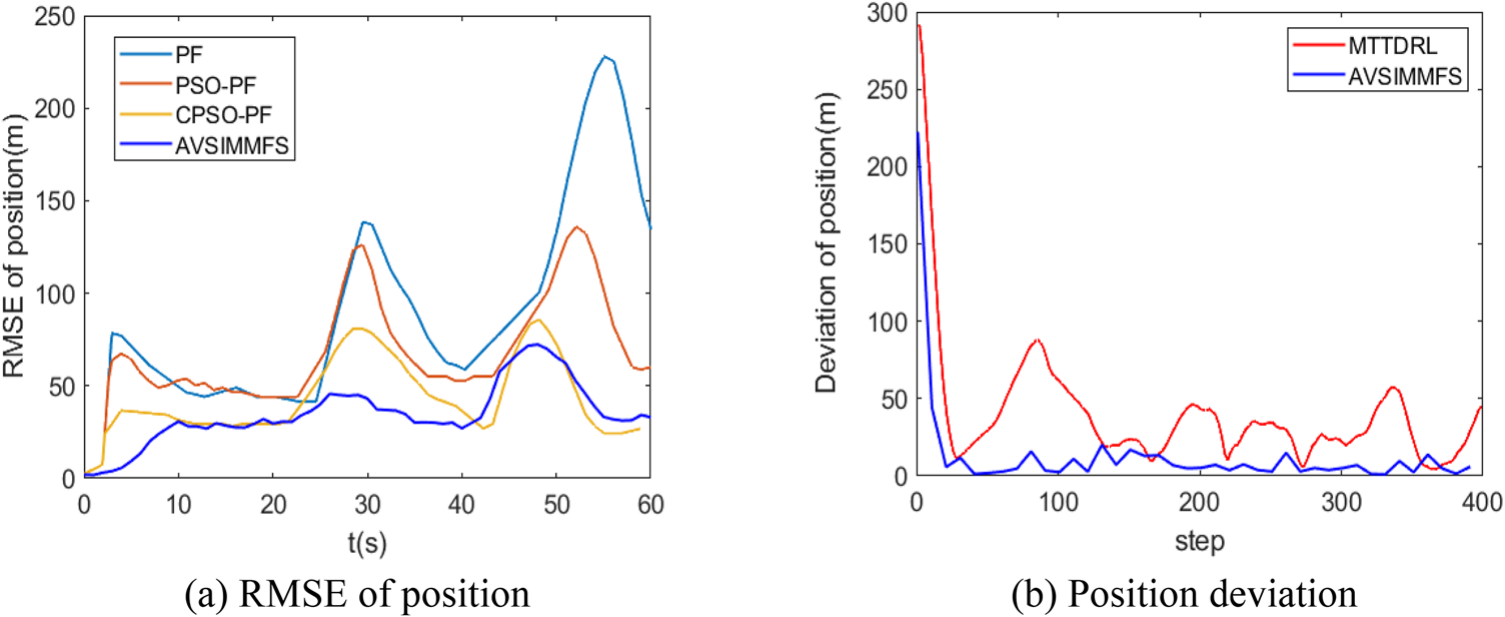
Performance comparison of algorithms.

To further demonstrate the superiority of AVSIMMFS algorithm. VSIMMFS is compared with Maneuvering Target Tracking based on Deep Reinforcement Learning (MTTDRL)^31^, Particle Filter (PF), Particle Swarm Optimization algorithm-based PF (PSO-PF)^32^, Chaos PSO-PF (CPSO-PF)^33,34^. In order to ensure the validity of the comparison, the simulation parameters are designed according to^31,34^, and the trajectories of the target is shown in Fig. 14. Figure 15 analyzes and compares the tracking performance of the above algorithm. It can be seen from Fig. 15a that the RMSE of AVSIMMFS is smaller than that of PF, PSO-PF and CPSO-PF. As can be seen from Fig. 15b, compared with MTTDRL algorithm, AVSIMMFS has smaller position deviation when tracking the target. In conclusion, AVSIMMFS has better tracking performance than other algorithms mentioned above.

The simulations of AVSIMMFS are implemented in a MATLAB environment, and the main configuration of the computer is 2.1 GHz CPU and 16 GByte RAM. When the smoothing interval is 150 steps, the time required for the smoothing process is about 0.06 s. Therefore, computers with better hardware performance will greatly improve the real-time performance of the algorithm. Under the same hardware configuration and smoothing step size, the computational cost comparison between the proposed algorithm and the existing algorithms is shown in Table 2. In terms of computation time and memory footprint, AVSIMMFS has the lowest computation cost: the memory footprint is in the median of existing algorithms, but the computation time is significantly less. Combined with the significant performance advantages, AVSIMMFS is clearly the best overall performance.

**Table 2:** Comparative analysis of algorithm resource consumption.

	AVSIMMFS	MTTDRL	PF	PSO-PF	CPSO-PF
Average time consuming	0.060 s	0.180 s	0.493 s	0.236 s	0.221 s
Memory footprint	320 MB	431 MB	94 MB	249 MB	397 MB

## Conclusion

In order to improve the target tracking performance, an AVSIMMFS algorithm is presented in this paper. This method builds a new model subset on the basis of the original model subset, which can improve the matching degree between the model subset and the actual maneuvering model of the target without excessively increasing the computational cost, so as to improve the tracking accuracy of the target. Finally, the filtering data of the AVSIMM algorithm is smoothed to obtain the AVSIMMFS algorithm, which further improves the target tracking accuracy. By using real data and simulation data to compare with IMM, normal VSIMM, AVSIMM, PF, PSO-PF, CPSO-PF and MTTDRL algorithms, AVSIMMFS has the best tracking performance. Compared with IMM and normal VSIMM, AVSIMMFS can construct a new model subset based on the original model subsets, which improves the matching accuracy of the model subset, thus improving the tracking accuracy. Due to the smoothing of the filtered data, the root mean square error of AVSIMMFS for position tracking is less than that of AVSIMM. In addition, compared with other types of filtering algorithms such as PF, PSO-PF, CPSO-PF and MTTDRL, AVSIMMFS also has the smallest tracking error. In terms of calculation cost, AVSIMMFS only needs about 0.06 s to obtain the results, showing good real-time performance.

## Data Availability

The datasets generated and/or analysed during the current study are not publicly available due the Chinese military’s strictest secrecy policy on hypersonic missile technology. but are available from the corresponding author on reasonable request.
